# KAT6A, a novel regulator of β-catenin, promotes tumorigenicity and chemoresistance in ovarian cancer by acetylating COP1

**DOI:** 10.7150/thno.57455

**Published:** 2021-04-15

**Authors:** Wenxue Liu, Zhiyan Zhan, Meiying Zhang, Bowen Sun, Qiqi Shi, Fei Luo, Mingda Zhang, Weiwei Zhang, Yanli Hou, Xiuying Xiao, Yanxin Li, Haizhong Feng

**Affiliations:** 1 State Key Laboratory of Oncogenes and Related Genes, Renji-Med X Clinical Stem Cell Research Center, Ren Ji Hospital, Shanghai Cancer Institute, School of Medicine, Shanghai Jiao Tong University, Shanghai 200127, China.; 2 Key Laboratory of Pediatric Hematology and Oncology Ministry of Health, Department of Hematology & Oncology, Shanghai Children's Medical Center, Shanghai Jiao Tong University School of Medicine, Shanghai, 200127, China.; 3 Department of Clinical Nutrition, Shanghai Children's Medical Center, School of Medicine Shanghai Jiao Tong University, Shanghai, 200127, China.; 4 Department of Obstetrics and Gynecology, Renji Hospital, School of Medicine, Shanghai Jiao Tong University, Shanghai 200127, China.; 5 Department of Radiotherapy, Renji Hospital, School of Medicine, Shanghai Jiao Tong University, Shanghai 200127, China.; 6 Department of Oncology, Renji Hospital, School of Medicine, Shanghai Jiao Tong University, Shanghai 200127, China.

**Keywords:** KAT6A, ovarian cancer, acetylation, COP1, β-catenin

## Abstract

**Background:** Ovarian cancer is a fatal gynecologic malignancy that is found worldwide and exhibits an insidious onset and a lack of early warning symptoms. Despite ongoing studies, the mechanistic basis of the aggressive phenotypes of ovarian cancer remains unclear. Lysine acetyltransferase 6A (KAT6A) is a MYST-type histone acetyltransferase (HAT) enzyme identified as an oncogene in breast cancer, glioblastoma and leukemia. However, the specific functions of KAT6A in ovarian cancer remain unclear.

**Methods:** Immunohistochemistry (IHC) staining and western blotting were performed to characterize KAT6A protein expression in ovarian cancer tissues and cell lines. The biological functions of KAT6A in ovarian cancer were evaluated by cell proliferation, wound healing and transwell invasion assays *in vitro*. Tumorigenesis and metastasis assays were performed in nude mice to detect the role of KAT6A *in vivo*. Mass spectrometry and immunoprecipitation assays were performed to detect the KAT6A-COP1 interaction. An *in vivo* ubiquitination assay was performed to determine the regulation of β-catenin by KAT6A.

**Results:** In the present study, we revealed that KAT6A expression is upregulated in ovarian cancer and is associated with patient overall survival. Downregulation of KAT6A markedly inhibited the proliferation and migration abilities of ovarian cancer cells *in vivo* and *in vitro*. Additionally, the inhibition of KAT6A induced apoptosis and enhanced the sensitivity of ovarian cancer cells to cisplatin. Furthermore, KAT6A bound to and acetylated COP1 at K294. The acetylation of COP1 impaired COP1 function as an E3 ubiquitin ligase and led to the accumulation and enhanced activity of β-catenin.

**Conclusions:** Our findings suggest that the KAT6A/COP1/β-catenin signaling axis plays a critical role in ovarian cancer progression and that targeting the KAT6A/COP1/β-catenin signaling axis could be a novel strategy for treating ovarian cancer.

## Introduction

Ovarian cancer (OC) remains one of the most lethal gynecological malignancies and the fifth leading cause of cancer-related death in female patients 1. In 2018, an estimated 295,414 new cases of ovarian cancer occurred worldwide, causing 184,799 deaths 2. Due to the lack of early warning symptoms and screening strategies, approximately 75% of cases are typically diagnosed at an advanced stage 3,4. Currently, cytoreductive surgery combined with platinum-based chemotherapy is the standard therapy. The majority of ovarian cancer patients respond to the first application of platinum-based chemotherapy. However, many patients develop drug resistance and experience relapse 5-7. Poly-ADP ribose polymerase inhibitors (PARPis) have been applied for maintenance therapy and benefit not only patients with deleterious germline or somatic BRCA1 or BRCA2 mutations but also patients with other forms of homologous recombination deficiency (HRD). Regrettably, many patients acquire resistance to PARPis that is similar to platinum resistance 8. The efficacy of immune checkpoint inhibitors (such as anti-PD-1) in the treatment of ovarian cancer is not ideal 9. Therefore, the exploration of innovative therapeutic strategies for precision therapy is urgently needed.

Lysine acetyltransferase 6A (KAT6A, also called MOZ and MYST3) is a histone acetyltransferase (HAT) belonging to the MYST family 10-11. KAT6A is composed of a nuclear localization domain, a double C2H2 zinc-finger domain that binds to acetylated histone tails, a HAT domain, a glutamate/aspartate-rich region, and a serine and methionine-rich transactivation domain 12. KAT6A has been reported to play a critical role in hematopoietic stem cell maintenance 13, cell cycle regulation 14, and cell senescence 15. In addition, KAT6A is thought to be an oncogene in human cancers, including breast cancer 16, glioma 17 and leukemia 18-20. KAT6A has been reported to not only catalyze the acetylation of histones 11, but also acetylate nonhistone proteins. However, to date, only p53 has been identified as a KAT6A nonhistone protein acetylation substrate 21. KAT6A inhibitors have been reported, and the inhibition of KAT6A shows promise for cancer therapy 22. Therefore, the mechanism by which KAT6A dysregulation contributes to tumorigenesis in some solid tumors, such as ovarian cancer, needs to be elucidated.

Here, to confirm the oncogenic role of KAT6A in ovarian tumorigenesis and the therapeutic potential of KAT6A inhibition, we performed a series of functional experiments *in vitro* and *in vivo*. Our study showed that KAT6A is overexpressed in ovarian cancer and associated with prognosis. Moreover, KAT6A, acting as a regulator of β-catenin, promotes the proliferation, invasion and metastasis of ovarian cancer cells and endows them with resistance to platinum-based chemotherapeutics by acetylating constitutive photomorphogenic 1 (COP1).

## Materials and Methods

### Cell lines and cell culture

SKOV3, Hey and A2780 cells were obtained from American Type Culture Collection (ATCC, USA). IOSE80, HO8910 and HO8910-PM cells were kindly provided by Stem Cell Bank, Chinese Academy of Sciences. HEK293T and Hela cells were maintained in our lab. All cell lines were cultured in Dulbecco's modified Eagle's medium (DMEM) supplemented with 10% fetal bovine serum (FBS) and maintained at 37 °C in 5% CO_2_. All cell lines were found to be mycoplasma-free and authenticated using short tandem repeat (STR) DNA fingerprinting at Shanghai Biowing Applied Biotechnology Co., Ltd. (Shanghai, China).

### Antibodies and reagents

The following antibodies were used in this study: anti-KAT6A [1:1000 for Western blot (WB), 1:50 for immunohistochemical (IHC) staining, 1:100 for immunoprecipitation (IP), Abnova]; anti-Ki-67 (1:500 for IHC, 1:2000 for flow cytometry, Invitrogen); PCNA (1:5000 for WB, Abcam), anti-Flag (1:1500 for WB, 1:100 for IP, Sigma-Aldrich); anti-HA (1:1000 for WB, 1:100 for IP, CST); anti-Ub, anti-Histone H3 (1:1000 for WB, CST); anti-STAT3, acetyl-Histone H3 (Lys23), acetyl-Histone H3 (Lys14), and acetyl-Histone H3 (Lys9), anti-acetylated lysine (1:1000 for WB, CST); anti-COP1 (1:1000 for WB, 1:200 for IF, 1:100 for IP, Abcam); anti-β-catenin (1:5000 for WB, 1:250 for IF, Abcam); anti-c-Myc, anti-CyclinD1, anti-p27 (1:1000 for WB, Proteintech), anti-p53 (1:5000 for WB, Abcam) anti-ETS2 (1:3000 for WB, Abcam), anti-c-JUN (1:2000 for WB, Abcam), anti-cleaved caspase-3, c-PARP (1:1000 for WB, CST), anti-Puma (1:3000 for WB, Abcam), anti-Bcl2 (1:1000 for WB, Abcam), anti-Bcl-XL (1:1000 for WB, Abcam) and anti-β-actin, anti-GAPDH, (1:1000 for WB, Proteintech). Cisplatin and CHX were obtained from Med Chem Express. WM-1119 and MG132 were obtained from Selleck. Wnt3a was obtained from R&D systems.

### Plasmids

The coding sequences of KAT6A, COP1 and β-catenin were amplified from the total cDNA of HEK293 cells, sequenced, and then subcloned into the pLenti-Blast vector 23 or pcDNA3.3 plasmid (Clontech). The coding sequences of adenomatous polyposis coli (APC) from DNA core in Shanghai Jiao Tong University were amplified, sequenced, and then subcloned into a pLenti-Blast or pcDNA3.3 vector. Taking advantage of the degeneracy of codons, shKAT6A or shCOP1-resistant overexpression plasmids were designed by replacing several codons in shRNA target sites with other codons coding the same amino acids. Point mutations were generated using a Site-Directed Mutagenesis Kit (Invitrogen) following the manufacturer's protocol.

### Immunohistochemistry

In line with a protocol approved by the Shanghai Jiao Tong University Institutional Clinical Care and Use Committee, clinical ovarian tissue samples were collected at Ren Ji Hospital, School of Medicine, Shanghai Jiao Tong University (Shanghai, China) according to the Declaration of Helsinki. Written informed consent was obtained from all patients. These samples were examined and diagnosed by pathologists at Ren Ji Hospital. The tissue sections were stained with antibodies against KAT6A (1:25) and Ki67 (1:200). IHC staining was scored as 0-7 according to the percentage of positive cells. Two independent pathologists who were blinded to the slides examined and scored each sample as follows: 7, strong staining in ~50% of tumor cells; 6, weak staining in ~50% of tumor cells; 5, strong staining in ~25% of tumor cells; 4, weak staining in ~25% of tumor cells; 3, strong staining in ~5 to 25% of tumor cells; 2, weak staining in ~5-25% of tumor cells; 1, low or no staining in < 1% of tumor cells; and 0, no detectable staining in any tumor cell (0%). Samples with staining scores of 0-2 were considered low expression, while those with scores of 3-7 were considered high expression.

### Cell proliferation assay

A Cell Counting Kit-8 (CCK-8; Yeasen, Shanghai) was used to perform cell proliferation assays according to the manufacturer's protocol.

### Colony formation assay

In brief, 500 of the indicated cells were plated into 6-well plates. Fourteen days later, colonies were fixed with methyl alcohol and stained with crystal violet. Then, the colonies were counted. All analyses were performed in triplicate.

### Transwell invasion assay

Cell migration assays were performed using 24-well Boyden chamber transwell inserts (BD Biosciences, USA). Corning Matrigel at a concentration of 200 μg/mL was added to the upper chamber. Cells (1 × 10^5^ in 200 μL of serum-free DMEM) were added to the upper chambers. The lower chamber of each well contained 600 μL of DMEM supplemented with 15% FBS. After incubation in an incubator at 37 °C in 5% CO_2_ for 24 h, cells that migrated to the bottom of the upper chamber membrane were stained with crystal violet and counted. All assays were repeated in triplicate.

### Wound healing assay

Cells were plated into 6-well plates. When the cells reached confluence, we gently scratched the monolayer with a pipette tip to create a mechanical wound. A microscope was used for imaging at 0 and 48 h. The experiment was performed in triplicate. Cell migration was compared by calculating the gap size in each field. The scratch area was measured using ImageJ. Average scratch width= scratch area/ length. Relative width = 48 h scratch width/0 h scratch width.

### Apoptosis assays

Ovarian cancer cells were transfected with Non-Target short hairpin RNA (shRNA) or KAT6A shRNA and cultured for 48 h. An Annexin V-APC Apoptosis Detection Kit (Invitrogen) was used according to the manufacturer's instructions. Fluorescence was detected using a BD FACSCanto flow cytometer (Becton Dickinson, Mountain View, CA, USA) and analyzed using FlowJo software (version 7.6.1). The experiment was performed in triplicate.

### Mass spectrometric analyses

Proteomics analyses for KAT6A-associated proteins and COP1 acetylation were performed at Jiyun Biotech. Inc. (Shanghai, China). Briefly, SKOV3 cells transfected with Flag- (His)_6_-tagged KAT6A or an empty vector control were lysed and purified using a Ni+-NTA column. Then, the protein samples were analyzed by LC-MS/MS using Q Exactive Plus (Thermo). The raw data were processed by MAXQUANT software. The raw data were searched against the UNIPROT database.

### Immunoprecipitation assays and western blot analysis

Harvested cells were lysed in IP lysis buffer (20 mM Tris-HCl pH 7.5, 150 mM NaCl, 1 mM EDTA, 2 mM Na3VO4, 5 mM NaF, 1% Triton X-100) supplemented with protease inhibitor cocktail (Invitrogen) at 4 °C for 30 min. Then, the lysates were cleared by centrifugation at 12,000 × g for 10 min at 4 °C. The supernatant was immunoprecipitated with the indicated antibodies and protein G-agarose beads (Invitrogen). Then, standard WB analysis was performed. For WB analysis, cells were lysed in radioimmunoprecipitation assay (RIPA) buffer (Beyotime Biotechnology, Jiangsu, China). Equal amounts of protein samples were separated by sodium dodecyl sulfate-polyacrylamide gel electrophoresis (SDS-PAGE) and then transferred onto polyvinylidene fluoride (PVDF) membranes (Millipore). The membranes were blocked in 5% milk for 2 h at room temperature and then incubated with the indicated primary antibodies overnight at 4 °C and HRP-conjugated secondary antibodies for 1 h at room temperature. The membranes were detected and analyzed using an infrared imaging system (Bio-Rad Laboratories, Inc.). The experiments were performed in triplicate.

### Single-guide RNA (sgRNA)-knockout, shRNA knockdown and transfection

Sequences of sgRNAs were designed using the MIT online tool (http://crispr.mit.edu) and cloned into a lentiCRISPRv2 vector. The guide RNA targeting sequences of KAT6A is TTCACTCGAACCGTTAGTTC. The guide RNA targeting sequences of COP1 is AGCCCAACTACAGATTCTTA. KAT6A and COP1 shRNAs were purchased from GeneChem, Inc. (Shanghai, China). Certain DNAs and packaging plasmids [pMD2. G (Addgene #12259) and psPAX2 (Addgene #12260)] were transfected into HEK293T cells using Hieff Trans^TM^ Liposomal Transfection Reagent (Yeasen) following the manufacturer's instructions. Forty-eight hours after transfection, virus-containing supernatants were collected, concentrated, and transduced into various cells.

### *In vivo* ubiquitination assays

Cells were transfected with combinations of plasmids, including His-ubiquitin plasmids. Forty-eight hours later, cells were treated with 10 μg/ml MG132 (Selleck) for 6 h. Subsequently, IP assays and WB analysis were performed as described above to detect the ubiquitination level of target proteins.

### RNA extraction and qRT-PCR

Total RNA was isolated from ovarian cancer cells with TRIzol (Thermo Fisher Scientific). cDNA was reverse transcribed with a Reverse Transcription Kit (Takara) according to the manufacturer's protocol. Quantitative PCR was performed with Power SYBR Green Master Mix (Life Technologies). The mRNA expression results were analyzed using the 2-(*ΔΔ*Ct) method.

Primers are listed as follows: *CCND1*, 5′-CCGCACGATTTCATTGAACACT-3′ and 5′-CGAAGGTCTGCGCGTGTTT-3′; *c-Myc*, 5′-GGACCTTCTGACCACGAT-3′ and 5′-GCAACAGCATAACGCCTC-3′; *MMP7*, 5′-AAGTGGTCACCTACAGGATCG-3′ and 5′-TGGCCCATCAAATGGGTAGG -3′; *ACTB*, 5′-CATGTACGTTGCTATCCAGGC-3′ and 5′-CTCCTTAATGTCACGCACGAT-3′.

### Animal experiments

Animal experiments were performed in adherence to guidelines approved by the Institutional Animal Care and Use Committee (IACUC) of Shanghai Jiao Tong University. A total of 1×10^6^ shKAT6A or control A2780 cells were subcutaneously inoculated into the hind flanks of 5-week-old female nu/nu mice. The lengths and widths of the tumors were measured every 5 days, and the tumor volume was calculated according to the following formula: volume = (length × width^2)/2.

For metastasis assays, we injected 1 × 10^6^ cells into the abdominal cavity of mice. After 4 weeks, the mice were sacrificed, and the numbers of metastatic foci were calculated. For analysis of the *in vivo* effects of cisplatin and WM-1119, A2780 cells were injected subcutaneously into the hind flanks of female nu/nu mice; cisplatin and WM-1119 were intraperitoneally injected into mice 7 days after tumor cell inoculation at 5 mg/kg and 60 mg/kg mouse body weight, respectively. Cisplatin was injected every three days for 21 days, and WM-1119 was injected 4 times per day.

### Statistical analysis

All experiments were performed independently at least three times. GraphPad Prism 8.0 software (San Diego, CA, USA) was used for statistical analysis. All data are presented as the mean ± standard deviation (SD) values from triplicate experiments. A P value of < 0.05 was considered significant. Differences between two groups were analyzed by independent samples *t*-tests; differences among multiple groups were analyzed by one-way ANOVA.

## Results

### KAT6A is upregulated in ovarian cancer and associated with a poor prognosis

**Figure 1:**
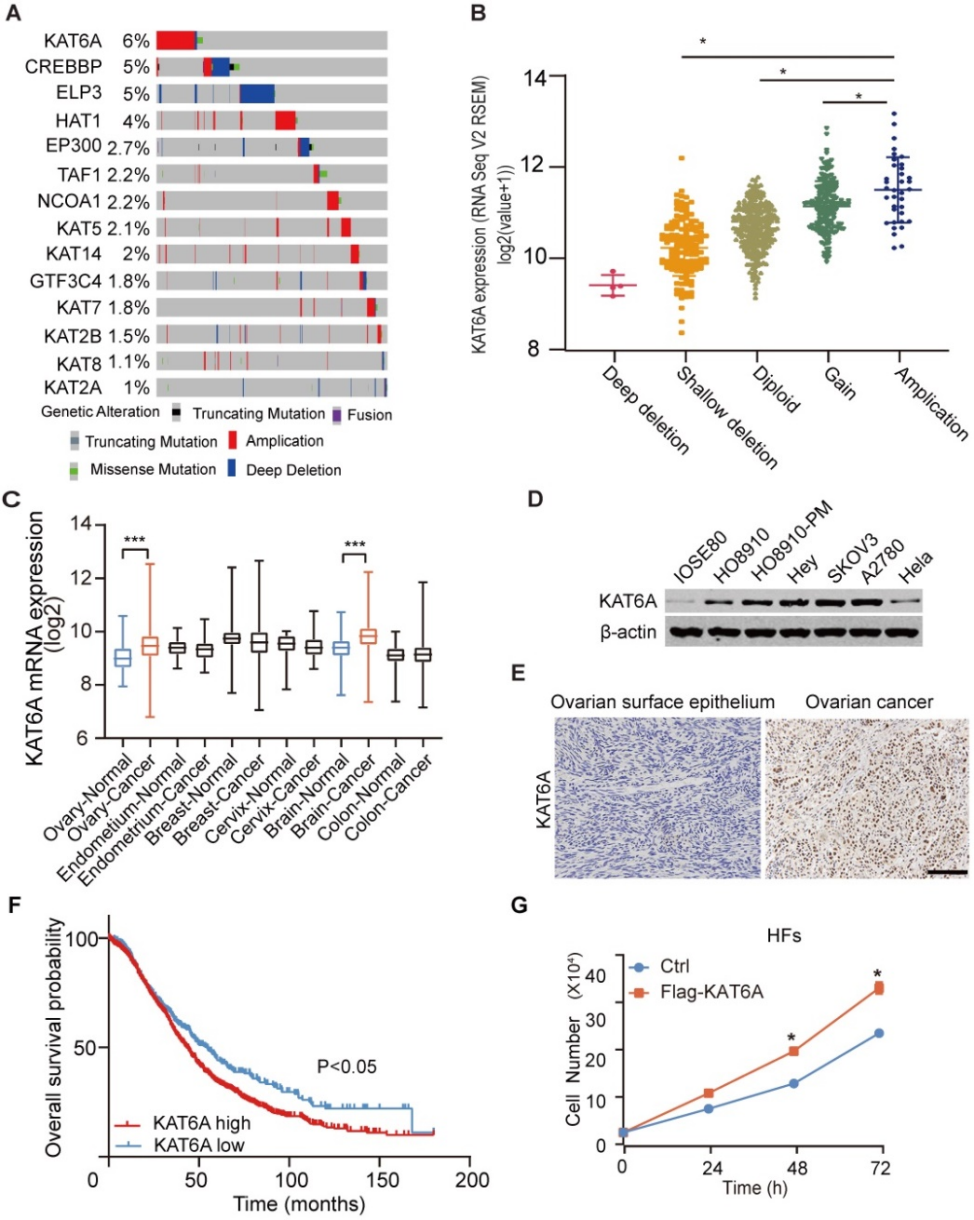
**KAT6A overexpression is associated with a poor overall survival in ovarian cancer. (A)** DNA copy number variation (CNV) of lysine acetyltransferase (KAT) genes, including KAT6A, in ovarian cancer in the TCGA dataset (https://www.cbioportal.org) (the red bar indicates copy number amplification; the gray bar indicates a normal copy number, and the blue bar indicates deep deletion). **(B)** KAT6A amplification is associated with its elevated mRNA expression in The Cancer Genome Atlas (TCGA) dataset of ovarian cancer. The data were from https://www.cbioportal.org. **(C)** KAT6A mRNA expression in the GENT2 datasets. **(D)** Western blotting (WB) analysis of KAT6A protein expression in ovarian cancer cells (SKOV3, A2780 and HEY), HO8910, HO8910-PM, Hela and normal ovarian epithelium cells (IOSE80). **(E)** Representative images of KAT6A immunohistochemical (IHC) staining in normal ovarian tissues and ovarian cancer tissues. **(F)** Overall survival of ovarian cancer patients with high (n=1209) vs. low (n=447) mRNA expression levels of KAT6A (216361_s_at, auto select best cut off), as determined by Kaplan-Meier survival analysis. **(G)** Cell proliferation was measured by cell counting in HFs with or without KAT6A overexpression. Scale bars: 50 µm. *P < 0.05, **P < 0.01.

To study the potential role of KAT6A in ovarian cancer, we first analyzed its copy number alterations in The Cancer Genome Atlas (TCGA). As shown in Figure 1A, KAT6A was generally amplified in ovarian cancer. KAT6A amplification was associated with elevated mRNA expression (Figure 1B). Amplified genomic KAT6A was present in approximately 6% of ovarian cancer samples, which was higher than that of other ordinary acetyltransferases. Additionally, we searched GENT2 datasets (http://gent2.appex.kr/gent2/) and found that compared with normal tissues, KAT6A mRNA expression was upregulated in ovarian cancer tissues and brain cancer tissues but not other cancer types, such as endometrium cancer, breast cancer, cervical cancer or colon cancer (Figure 1C). To further support these findings, we examined KAT6A expression in ovarian cancer tissues and cell lines. The results suggested that KAT6A exhibited higher expression in ovarian cancer cell lines than in normal ovarian cell lines and Hela cells (Figure 1D). Moreover, KAT6A was highly expressed in 30 of 40 (75%) ovarian cancer tissues (Figure 1E and Table 1). Then, we analyzed Kaplan-Meier Plotter datasets (http://kmplot.com/) 24, and Kaplan-Meier survival analysis revealed that patients with high KAT6A expression had a shorter overall survival time than those with low KAT6A expression (Figure 1F). Taken together, these findings demonstrated that KAT6A is upregulated in ovarian cancer, and the overexpression of KAT6A may predict poor survival in ovarian cancer patients.

Additionally, based on previous research and our findings, we suspected that KAT6A may also exhibit oncogenic function in ovarian cancer. To preliminarily explore the function of KAT6A, we overexpressed KAT6A in human fibroblasts (HFs) and detected the effect of KAT6A on proliferation. The results showed that KAT6A overexpression promoted HF proliferation (Figure 1G), which indicated that KAT6A overexpression may play a tumor-promoting role in the development of cancer.

### Suppression of KAT6A inhibits the proliferation, invasion, and metastasis of ovarian cancer cells and suppresses ovarian tumor growth

**Figure 2:**
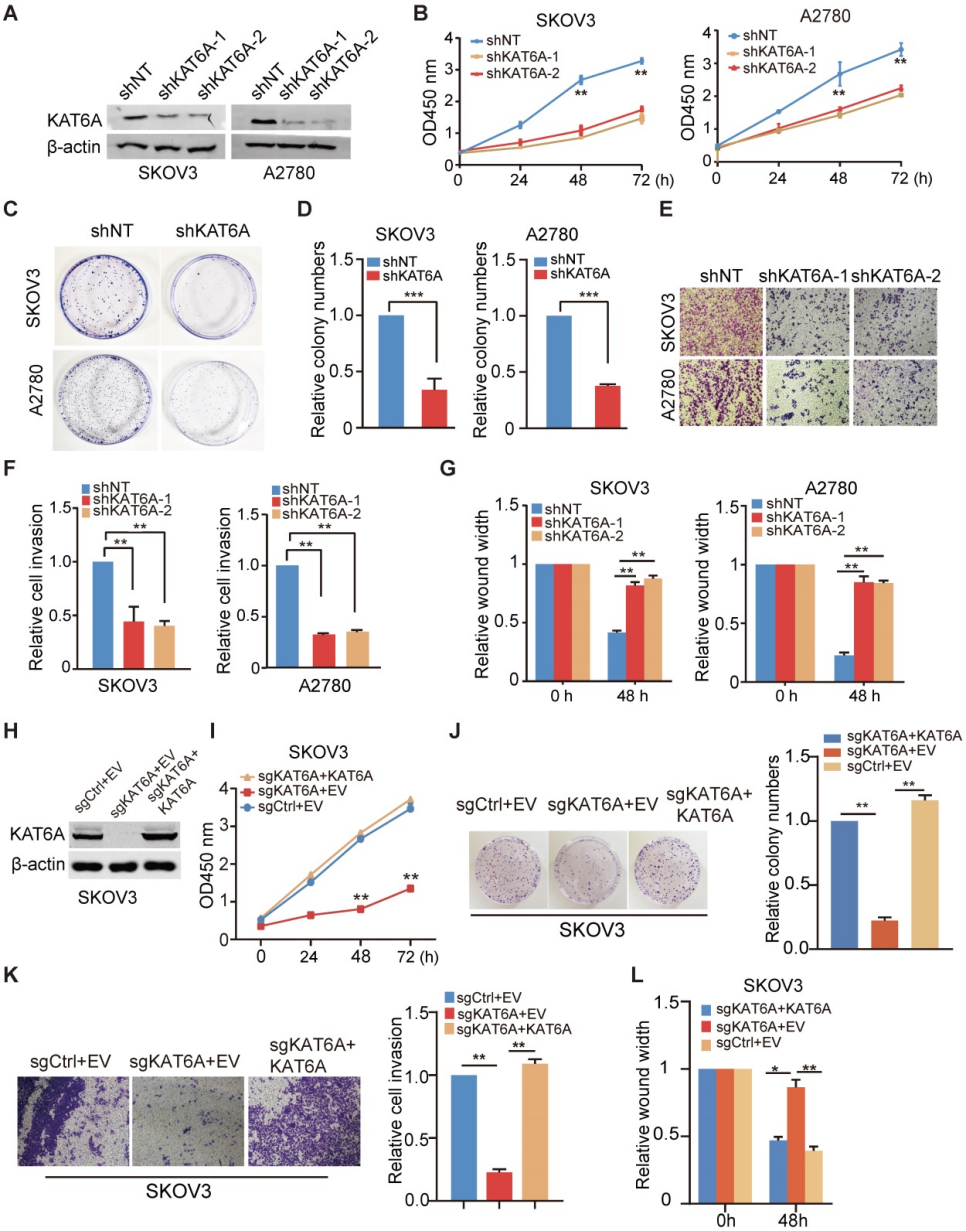
** Suppression of *KAT6A* inhibits the proliferation, invasion, and metastasis of ovarian cancer cells *in vitro*. (A)** Knockdown (KD) of *KAT6A* with two different shRNAs in SKOV3 and A2780 cells. **(B)** Cell proliferation was measured by CCK-8 assays in SKOV3 and A2780 cells with KAT6A knockdown. **(C and D)** Effects of KAT6A inhibition on the colony formation of SKOV3 cells and A2780 cells. **(E and F)** A transwell invasion assay was performed to evaluate the invasion ability of SKOV3 and A2780 cells. Representative images of migrated SKOV3 cells (up) and A2780 cells (down) in E. Quantification of migrated SKOV3 cells (left) and A2780 cells (right) in F. **(G)** A wound healing assay was performed to evaluate the migration ability of SKOV3 (left) and A2780 cells (right) with or without KAT6A knockdown. **(H)** Knockout (KO) of KAT6A with CRISPR/CAS9 in SKOV3 and KAT6A overexpression in KAT6A-KO SKOV3 cell line. **(I)** Cell proliferation was measured by CCK-8 assays in KAT6A-KO SKOV3 with or without rescue. **(J)** Effects of KAT6A KO on colony formation of SKOV3 cells. **(K)** Effects of KAT6A KO on invasion ability of SKOV3 cells. **(L)** Effects of KAT6A KO on the migration of SKOV3 cells. The data are presented as the mean ± SD. Statistical significance was assessed by a two-tailed Student's *t*-test or one-way ANOVA. *P < 0.05, **P < 0.01.

To further investigate the biological function of KAT6A in ovarian cancer, we knocked down KAT6A with two different KAT6A shRNAs in SKOV3 and A2780 cells and a non-target shRNA was used as a control (Figure 2A). As shown in Figure 2B, KAT6A silencing significantly impaired the proliferative ability in SKOV3 and A2780 cells. Moreover, depletion of KAT6A decreased colony formation in SKOV3 and A2780 compared with the control (Figure 2C-D). The efficiency of *KAT6A* knockdown (KD) in A2780 cells was higher than that in SKOV3 cells, but the effects of *KAT6A* KD on the proliferation of the two cell lines were similar. Due to differences in genetic and epigenetic backgrounds and in the basic phenotypes (cell cycle, apoptosis, senescence, etc.) of the cell lines, the response of different cells to certain treatments could be different.

Next, we hypothesized that KAT6A may play a role in the metastasis of ovarian cancer. We performed wound healing and transwell invasion assays and found that silencing *KAT6A* reduced the invasive ability in ovarian cancer cells (Figure 2E-F). Similarly, *KAT6A* KD significantly reduced cell migration (Figure 2G). These results demonstrate that KAT6A is important for ovarian cancer cell invasion and migration *in vitro*.

To confirm that KAT6A promotes the proliferation, migration and invasion in ovarian cancer cells, we knocked out *KAT6A* using CRISPR/Cas9 system in SKOV3 cells (Figure 2H). *KAT6A* knockout (KO) significantly reduced cell proliferation (Figure 2I), colony formation (Figure 2J), invasion ability (Figure 2K), and migration (Figure 2L) in SKOV3 cells. On the other hand, overexpression of KAT6A completely rescued the proliferation, migration and invasion ability of *KAT6A*-KO SKOV3 cells.

**Figure 3:**
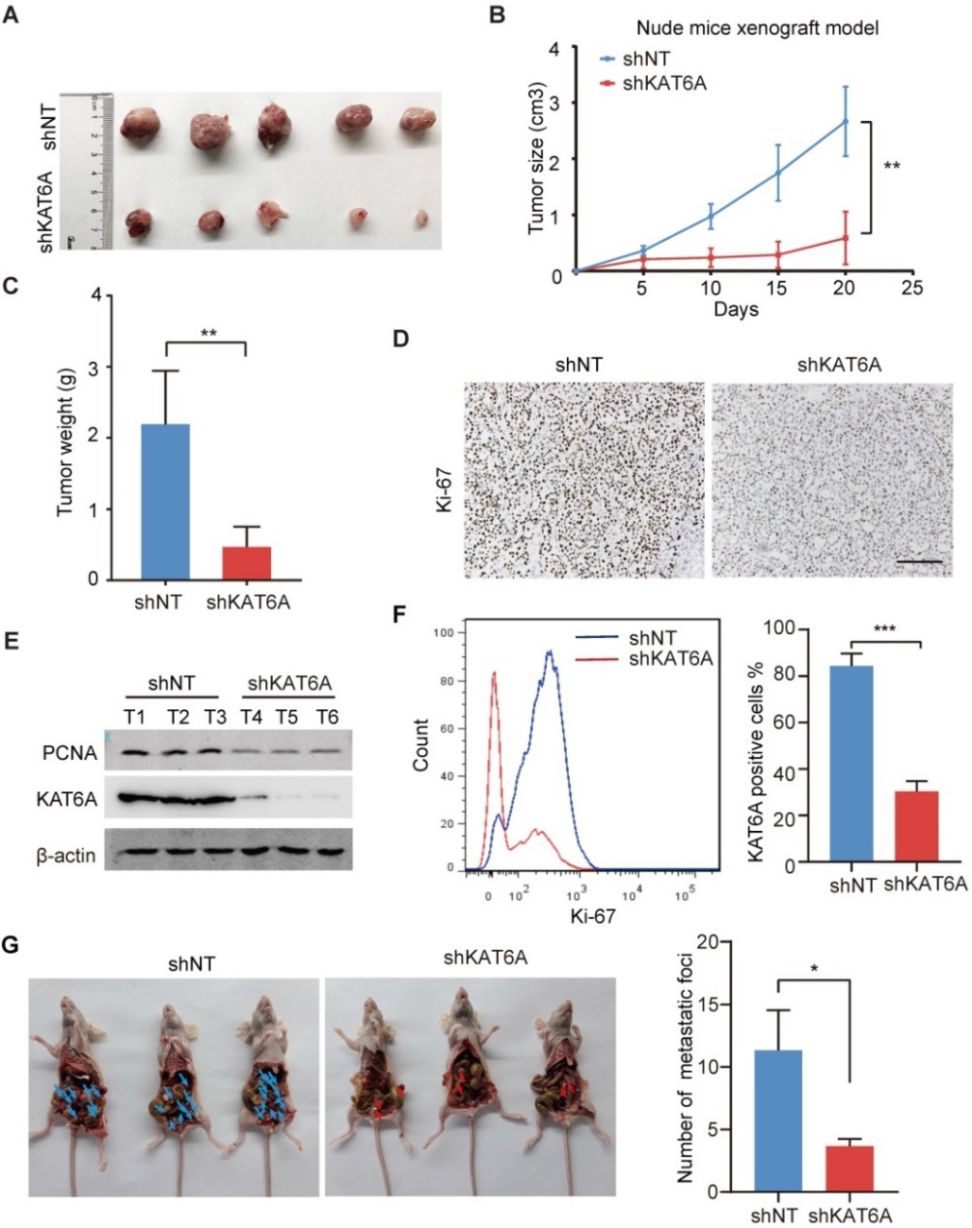
** Knockdown of KAT6A suppresses ovarian tumor growth *in vivo*. (A)** Representative images of KAT6A knockdown (down) A2780 xenografts relative to the control (up). **(B and C)** Tumor growth of the indicated A2780 cells in nude mice (n = 5 mice per group). Tumor volumes (B) and tumor weights (C) were calculated. **(D)** The proliferation index Ki-67 was evaluated by IHC in xenografts. **(E)** WB analysis was performed to assess the expression of KAT6A and the proliferation index PCNA in xenograft tumors. **(F)** Flow cytometry was conducted to assess K*i*-67 in xenografts. **(G)** Representative images (left) and quantification (right) of abdominal cavity metastatic tumors derived from KAT6A-knockdown or control A2780 cells. The data are shown as the mean ± SD. *P < 0.05, **P < 0.01. Scale bar: 50 µm.

To further examine the oncogenic effects of KAT6A during ovarian tumorigenesis *in vivo*, we subcutaneously implanted A2780 cells into female nude mice. *KAT6A* KD markedly suppressed ovarian tumor growth (Figures 3A-C). In addition, subcutaneous xenografts of A2780 cells with KAT6A knockdown showed impaired proliferation compared to those with a control shRNA (Figure 3D-F). Next, to further study the role of KAT6A in the metastasis of ovarian cancer *in vivo*, KAT6A-silenced cells were injected into the abdominal cavity to develop a peritoneal metastasis model. Four weeks after inoculation, the numbers of metastatic nodules were counted. The results showed that inhibition of KAT6A significantly decreased the capacity of the cells to form secondary tumors in the abdominal cavity (Figure 3G). Collectively, these results demonstrate the oncogenic role of KAT6A in ovarian cancer by promoting cell proliferation and metastasis.

### KAT6A physically associates with COP1

**Figure 4:**
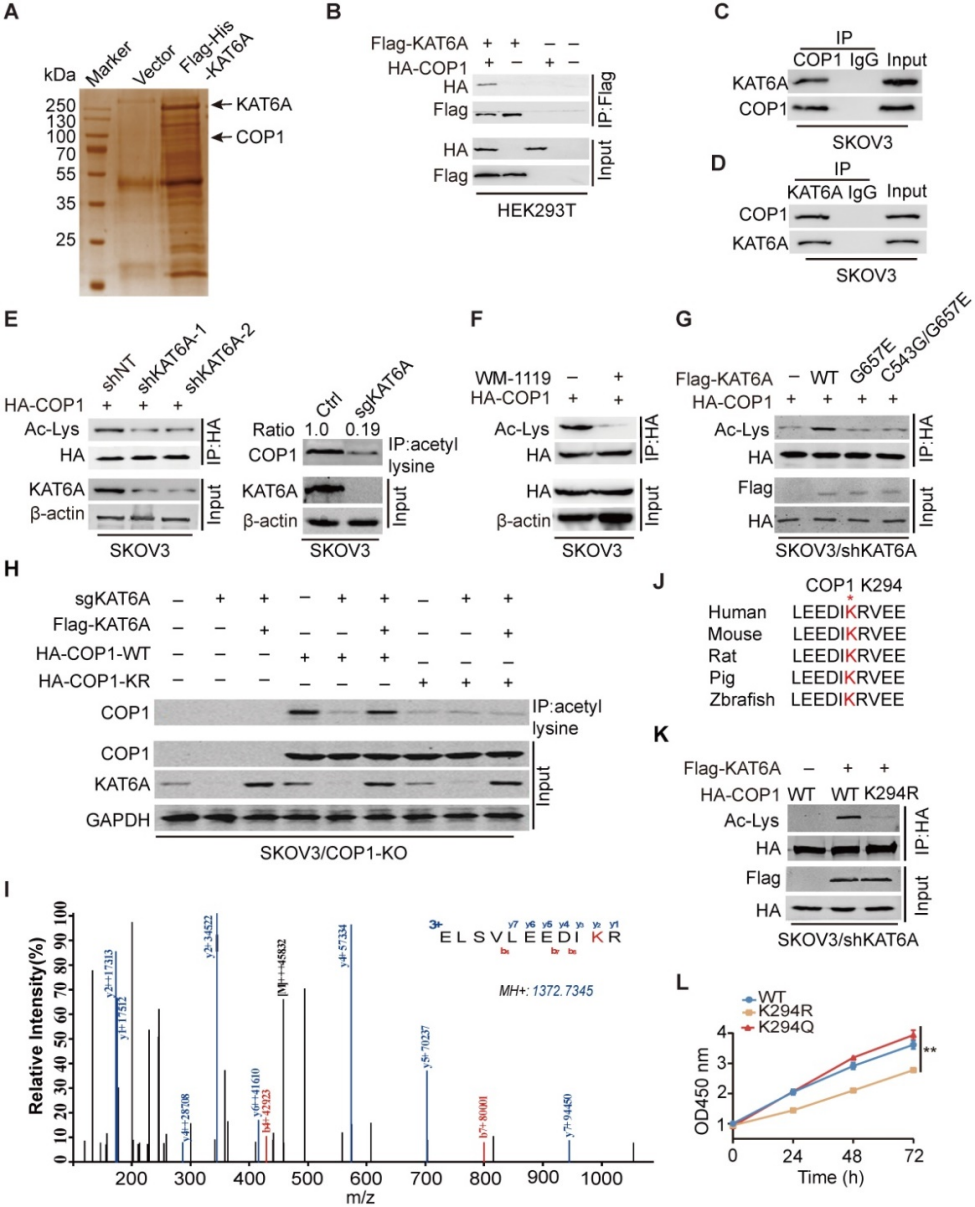
** KAT6A physically associates with COP1. (A)** Cellular extracts from SKOV3 cells expressing Flag-6xHis-KAT6A were pulled down with a Ni+-NTA column. The eluates were resolved by SDS/PAGE, subjected to silver staining, and analyzed by mass spectrometry. **(B)** Immunoprecipitation (IP) indicated that KAT6A interacted with COP1. HEK293T cells were co-transfected with plasmids expressing Flag-KAT6A and HA-COP1. **(C and D)** Immunoprecipitation analysis was performed to detect KAT6A binding with COP1. **(E)** Western blot analysis was conducted to assess the effect of KAT6A knockdown (left) or knockout (right) on COP1 acetylation in SKOV3 cells. **(F)** WB was performed to assess the effect of a KAT6A inhibitor (WM-1119, 25 µM) on COP1 acetylation in SKOV3 cells. **(G)** Effects of overexpression of shRNA-resistant KAT6A-WT or the G657E and C543G/G657E KAT6A mutants on COP1 acetylation. **(H)** Immunoprecipitation-western blot analysis for analyzing the effect of KAT6A knockout or COP1-K294R mutant on COP1 acetylation in SKOV3 (COP1-KO) cells. **(I)** Acetylation of COP1 by KAT6A was identified by liquid chromatography-tandem mass spectrometry (LC-MS/MS) analysis. **(J)** The amino acid sequences surrounding K294 in COP1 in multiple species. **(K)** Effect of KAT6A on the acetylation of COP1-WT or COP1-K294R. **(L)** Effect of shRNA-resistant COP1-WT, K294R and K294Q mutants on SKOV3 proliferation ability.

We next investigated how *KAT6A* KD suppresses the development of ovarian tumorigenesis. As demonstrated by previous studies, KAT6A can acetylate both histones and nonhistone proteins 11,21. We found that KAT6A inhibition in ovarian cancer cells did not alter the acetylation level of several histones that are known to be substrates of KAT6A (Supplementary Figure S1) 25. To characterize the mechanism by which KAT6A promotes ovarian cancer, we performed mass spectrometry (MS) to identify KAT6A-binding partners and proteins acetylated by KAT6A. Among the top possible KAT6A-binding partners (Supplementary Table S1), COP1 was identified as acetylated by KAT6A (Figure 4A and 4I). COP1 (also called RFWD2) is an E3 ubiquitin ligase that has been reported to be overexpressed in ovarian cancer 26. To support that KAT6A binds to COP1, we performed immunoprecipitation and found that KAT6A interacted with COP1 (Figure 4B). The interaction between endogenous KAT6A and COP1 was confirmed in SKOV3 cells (Figure 4C-D). Then, we assessed the effect of KAT6A inhibition on COP1 acetylation with a pan-anti-acetylated lysine antibody. As shown in Figures 4E-F, inhibition of KAT6A downregulated COP1 acetylation by approximately 80% in SKOV3 cells. Next, HA-tagged COP1 and shKAT6A-resistant wild-type (WT) KAT6A or KAT-deficient mutants (KAT6A G657E or C543G/G657E) were ectopically co-expressed in SKOV3 cells with silencing of endogenous *KAT6A*, and the acetylation level of COP1 was assessed with a pan-anti-acetylated lysine antibody. As shown in Figure 4G, WT KAT6A rescued COP1 acetylation, whereas the KAT-deficient (KAT6A G657E or C543G/G657E) mutant did not. To confirm the COP1-ac band in Figures 4E-G, we generated a *COP1*-KO SKOV3 cell line using the CRISPR/Cas9 system. We found that COP1 could be immunoprecipitated by a pan-anti-acetylated lysine antibody, whereas the bands could not be detected in the *COP1* KO SKOV3 cell line (Figure 4H), indicating that COP1 is acetylated by KAT6A.

Next, we performed mass spectrometry analysis and identified that the lysine 294 (K294) residue of COP1 was acetylated by KAT6A (Figure 4I), which is highly conserved in COP1 among various species (Figure 4J). We then mutated K294 of COP1 to arginine (K294R, acetylation-dead mutant), which significantly impaired KAT6A-induced acetylation of COP1 in ovarian cancer cells (Figure 4H and 4K). Additionally, compared with COP1 WT or the K294Q mutant (acetylation mimic mutant), the K294R mutant repressed SKOV3 cell growth (Figure 4L). Collectively, these results indicated that KAT6A directly binds to and acetylates COP1 at K294, and the acetylation of COP1 K294 is important for ovarian cell proliferation.

### KAT6A stabilizes β-catenin by impairing its ubiquitination

**Figure 5:**
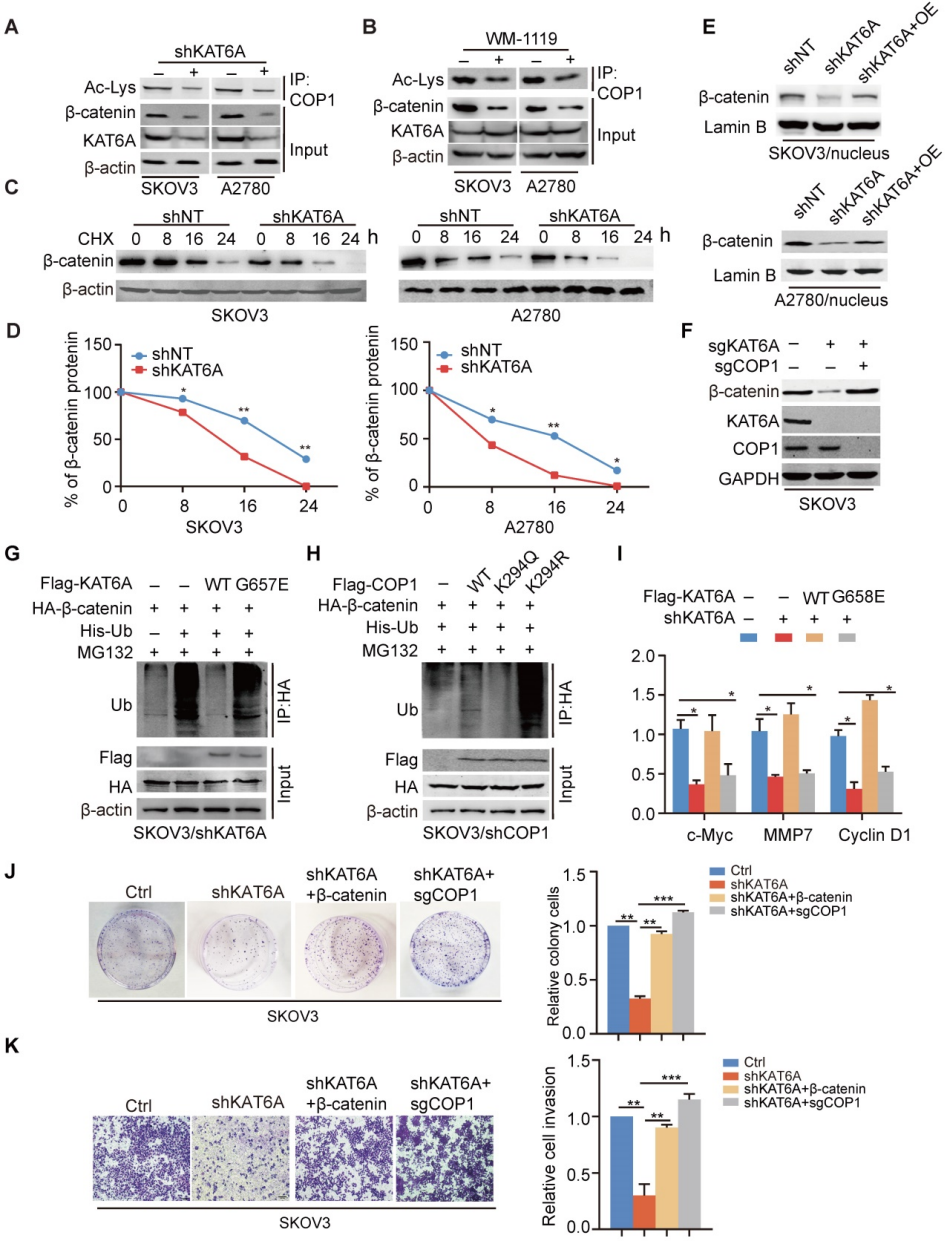
** KAT6A stabilizes β-catenin by impairing its ubiquitination. (A)** IP and Western blot analysis of the effects of KAT6A knockdown on COP1 acetylation and the β-catenin protein level in SKOV3 and A2780 cells. **(B)** IP and Western blot analysis of the effects of a KAT6A inhibitor (WM-1119, 25 µM) on COP1 acetylation and β-catenin protein levels in SKOV3 and A2780 cells. **(C)** Effects of KAT6A knockdown on β-catenin protein stability in SKOV3 (left) and A2780 (right) cells. **(D)** Quantification of β-catenin protein levels in C. **(E)** WB of the effects of KAT6A knockdown and the reverse effect of overexpression of KAT6A on the β-catenin protein level in the nucleus of SKOV3 and A2780 cells. **(F)** WB of the effects of KAT6A KO and COP1 KO on β-catenin protein levels. **(G)** Effects of KAT6A-WT and the acetyltransferase activity-deficient mutant KAT6A-G657E on β-catenin ubiquitination. His-Ub was co-transfected into SKOV3/shKAT6A cells with KAT6A constructs or empty vector control. **(H)** Effects of COP1 WT, K294R, and K294Q mutants on β-catenin ubiquitination. His-Ub was co-transfected into SKOV3/shCOP1 cells with COP1 constructs or empty vector control. **(I)** Effects of overexpression of shRNA-resistant KAT6A-WT or KAT6A-G657E mutant on CyclinD1, MMP7 and c-Myc mRNA expression levels.** (J and K)** The reverse effects of β-catenin overexpression or COP1 KO on cell colony formation (J) and cell invasion ability (K).

We further determine how COP1 acetylation contributes to the progression of ovarian cancer as an E3 ubiquitin ligase. We detected the effects of *KAT6A* KD on the levels of COP1-targeted proteins, including p53, p27, STAT3, ETS2, β-catenin, and c-JUN 27 in SKOV3 and A2780 cells. *KAT6A* KD or WM1119 treatment decreased β-catenin expression significantly (Figure 5A-B) but not that of COP1-targeted (Supplementary Figure 2A). Re-expression of COP1 K294R mutant did not influence COP1-targeted proteins (Supplementary Figure 2B) but decreased the protein level of β-catenin (Supplementary Figure 2C). β-catenin has been reported to be a substrate of COP1 28-29 and immoderate activation of Wnt/β-catenin signaling is involved in many malignancies, including ovarian cancer 30. Thus, according to our data above, we hypothesized that KAT6A regulates β-catenin protein stability by acetylating COP1. To test our hypothesis, we investigated the effects of KAT6A knockdown on the expression and protein half-life of β-catenin in SKOV3 and A2780 cells. Consistent with our hypothesis, repression of KAT6A reduced β-catenin protein level by downregulating COP1 acetylation (Figure 5A-B). KAT6A knockdown markedly decreased the stability of endogenous β-catenin in SKOV3 and A2780 cells (Figure 5C-D). We also found that knockdown of KAT6A significantly reduced the protein level of β-catenin in the nuclei of SKOV3 and A2780 cells and that the decreased the protein level of β-catenin in the nucleus was reversed by the overexpression of KAT6A (Figure 5E), which indicates that KAT6A may regulate the transcriptional activity of β-catenin. Next, we evaluated whether KAT6A regulates β-catenin protein levels in a COP1-dependent manner. Indeed, SKOV3 cells with KAT6A knockout markedly decreased the protein level of β-catenin compared to control cells (Figure 5F), while the effects on β-catenin protein level was abrogated with the co-depletion of COP1. To further determine whether the effect of KAT6A on β-catenin protein stability depends on its HAT activity, we assessed the ubiquitination level of β-catenin in cells expressing KAT6A-WT or the KAT-deficient G657E or C543G/G657E mutant. As shown in Figure 5G, compared with KAT6A-WT, the KAT-deficient mutants significantly increased the ubiquitination level of β-catenin.

Previous publications have identified APC as a negative regulator of β-catenin 31. We found that the overexpression of APC in SKOV3 cells decreased β-catenin protein levels, and the knockdown of KAT6A in APC-overexpressed cells further decreased the β-catenin level (Supplementary Figure 2D). Moreover, Wnt3a (100 ng/ml) increased the β-catenin protein level, while knockdown of KAT6A abrogated the upregulation of β-catenin (Supplementary Figure 2E). These results suggested that KAT6A may regulate β-catenin expression in a Wnt/APC independent manner.

Next, to determine whether the acetylation of COP1 influences the ubiquitination of β-catenin, we compared the effects of acetylation-mimic COP1(K294Q) and the acetylation-dead K294R mutant on β-catenin ubiquitination. As shown in Figure 5H, COP1-K294Q exhibited an impaired ubiquitination function and thereby attenuated β-catenin ubiquitination, whereas the COP1 K294R mutant enhanced β-catenin ubiquitination, suggesting that the acetylation of COP1 by KAT6A decreases β-catenin protein degradation and enhances β-catenin stability as well as β-catenin signal activation. In addition, the mRNA expression of cyclin D1, c-Myc and MMP-7, three downstream effectors of canonical Wnt/β-catenin signaling, was decreased by KAT6A knockdown and rescued by overexpression of shRNA-resistant KAT6A-WT but not the KAT-deficient mutant, which indicated that KAT6A could regulate the transcriptional activity of β-catenin (Figure 5I). To assess whether KAT6A promotes tumorigenesis of ovarian cancer through COP1-β-catenin pathway, we performed colony formation assay and transwell invasion assay. As shown in Figure 5J-K, overexpression of β-catenin rescued the abilities of proliferation and invasion which were impaired by KAT6A deficiency, and *COP1* knockout (KO) abrogated the effects of KAT6A knockdown in the abilities of proliferation and invasion, indicating that KAT6A promotes ovarian cancer progression through acetylating COP1 and stabilizing β-catenin. Taken together, these results demonstrate that the acetylation of COP1 by KAT6A stabilizes β-catenin by impairing β-catenin ubiquitination, resulting in the abnormal activation of the β-catenin signaling pathway and promoting the proliferation and metastasis of ovarian cancer cells.

### Inhibition of KAT6A induces apoptosis in ovarian cancer cells and enhances their sensitivity to cisplatin treatment

**Figure 6:**
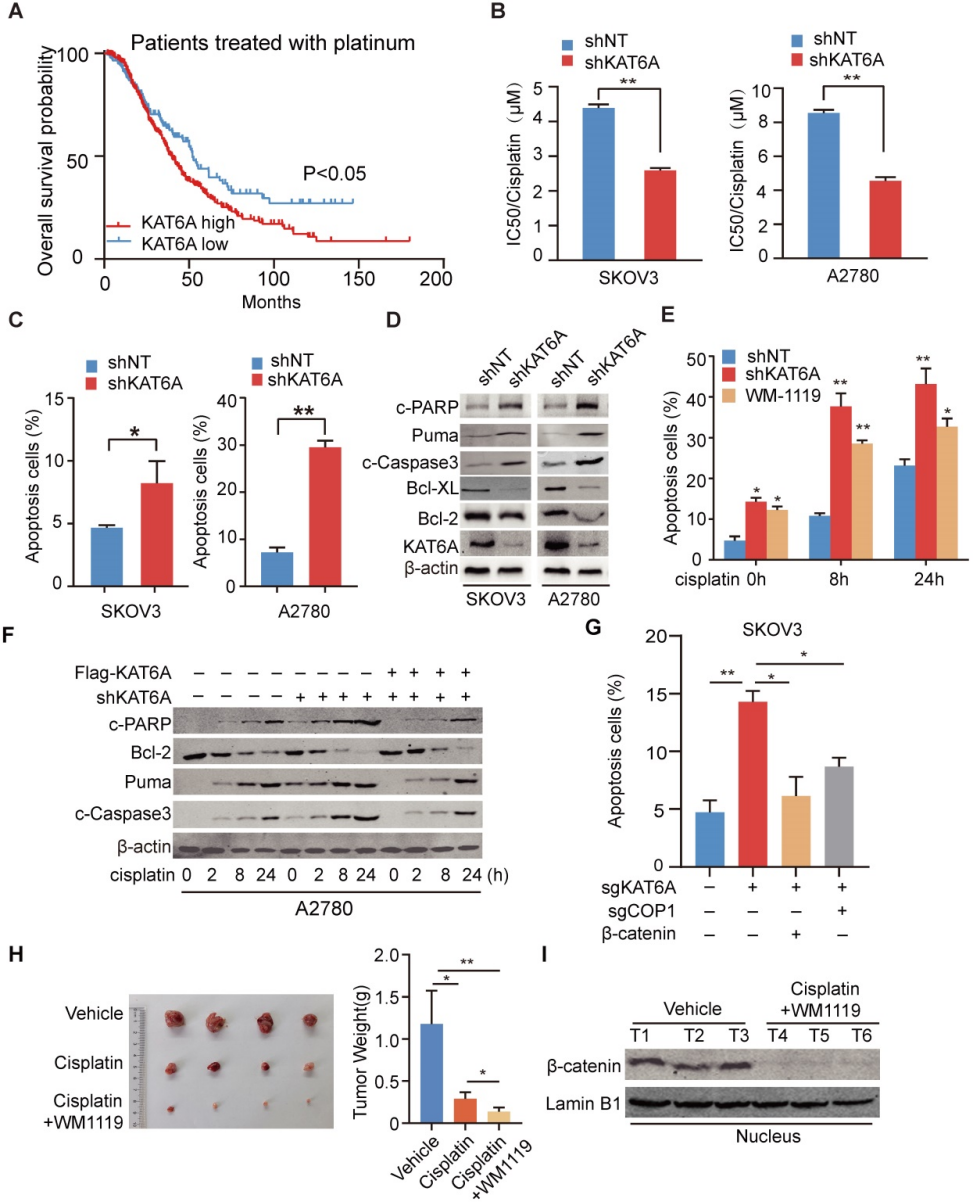
** Inhibition of KAT6A induces apoptosis in ovarian cancer cells and enhances their sensitivity to cisplatin treatment. (A)** Overall survival of patients with high (n=337) *vs*. low (n=141) levels of KAT6A (226547_at, auto select best cutoff, contains platin) mRNA expression among ovarian cancer patients treated with platinum-based agents, as determined by Kaplan-Meier survival analysis. **(B)** SKOV3 (left) and A2780 (right) cells with stable KAT6A knockdown were treated with different concentrations of cisplatin for 48 h, and cell viability was assessed by a Cell Titer-Glo (CTG) assay. **(C)** Flow cytometric histograms indicating apoptosis detection in SKOV3 (left) and (right) A2780 cells with KAT6A silencing. **(D)** The expression of apoptosis markers (Bcl2, Bcl-XL, Puma, c-PARP and c-Caspase3) in SKOV3 (left) and A2780 (right) cells with KAT6A knockdown was analyzed by WB. **(E)** Apoptosis analysis was performed at 0, 8 and 24 h after cisplatin treatment (2 µM) among shNT cells, shKAT6A cells and cells pre-treated with 25 µM KAT6A inhibitor (WM-1119) for 72 h. **(F)** The expression of apoptosis markers (Bcl2, Puma, c-Caspase3 and c-PARP) in A2780 cells with KAT6A knockdown or re-expression of KAT6A was detected by western blotting after treatment with cisplatin (2 µM). **(G)** Effects of overexpression of β-catenin or COP1-KO on KAT6A KO-induced apoptosis were assayed by flow cytometry. *P < 0.05, **P < 0.01. **(H)** A2780 cells were injected subcutaneously into the hind flanks of nu/nu mice; mice then received cisplatin and WM-1119 as described in the Methods. Tumor images (left) and weight (right) of isolated tumors are shown. **(I)** The expression of nuclear β-catenin in mice in the cisplatin and WM-1119 groups and the vehicle group.

We analyzed the Kaplan-Meier database (http://kmplot.com/) and found that among patients receiving platinum-based chemotherapy, patients with KAT6A overexpression had a poorer prognosis (Figure 6A). To determine whether KAT6A regulates the sensitivity of ovarian cancer cells to platinum-based chemotherapeutics, we measured the IC_50_ values of cisplatin in KAT6A-silenced cells and control cells. The IC_50_ values of cisplatin were 2.586 μM and 4.557 μM in KAT6A-silenced SKOV3 and A2780 cells, respectively, compared with 4.390 μM and 8.550 μM in the respective control cells (Figure 6B), suggesting that KAT6A knockdown increased the sensitivity of ovarian cancer cells to platinum-based chemotherapeutics. To further explore the mechanism by which KAT6A inhibition affects the sensitivity of ovarian cancer cells to platinum-based chemotherapeutics, we conducted an apoptosis assay by flow cytometry and found that inhibition of KAT6A in SKOV3 and A2780 cells increased cell apoptosis compared to that in the corresponding control cells (Figure 6C and Supplementary 3A). We also detected apoptosis-related marker protein expression and found higher expression levels of apoptosis-related proteins and lower expression levels of Bcl-2 and Bcl-XL in KAT6A-depleted SKOV3 and A2780 cells (Figure 6D). These data indicate that inhibition of KAT6A can induce apoptosis in ovarian cancer cells, enhancing their sensitivity to cisplatin treatment.

Given the above results, we were encouraged to explore the effects of KAT6A pharmacologic inhibition. We pre-treated A2780 cells with the KAT6A inhibitor WM-1119 to then subject them to flow cytometry to assess apoptosis. As shown in Figure 6E and Supplementary 3B, inhibition of KAT6A enhanced the cell apoptosis induced by cisplatin. However, the re-expression of KAT6A neutralized the influence of KAT6A inhibition on apoptosis in ovarian cancer cells (Figure 6F), indicating that KAT6A promotes ovarian cancer cell resistance to cisplatin treatment.

The apoptosis of SKOV3 cells induced by KAT6A knockout was rescued by the supplementation of Wnt3a and exacerbated by the overexpression of APC (Supplementary 3D), indicating that the inhibition of KAT6A cannot block the influence of Wnt/APC on apoptosis. Then, we evaluated whether KAT6A regulates apoptosis in a COP1-β-catenin dependent manner. Indeed, SKOV3 cells with KAT6A depletion exhibited markedly increased apoptosis compared with control cells, while the effects on apoptosis were abrogated by the co-depletion of COP1 (Figure 6G and Supplementary 3C). Notably, restoring β-catenin expression fully reversed the effects of inducing apoptosis in KAT6A-depleted SKOV3 cells (Figure 6G and Supplementary 3C). These results demonstrated that KAT6A regulates apoptosis by COP1 acetylation and stabling β-catenin, which is independent of Wnt/APC.

To further explore whether inhibiting KAT6A activity could be an effective therapeutic strategy in ovarian cancer patients with chemoresistance, mice with A2780 xenografts received an intraperitoneal injection of cisplatin and WM-1119 at 5 mg/kg and 60 mg/kg mouse body weight, respectively. As shown in Figure 6H, the combination of cisplatin and WM-1119 led to a significant reduction in tumor burden compared with the mice treated with cisplatin alone, and weight loss was not observed. Additionally, western blot analysis showed decreased nuclear β-catenin levels in tumor tissues of mice in the WM-1119 group (Figure 6I). Together, these data suggest that inhibition of KAT6A enhances cell apoptosis in ovarian cancer cells and sensitivity of ovarian cancer cell to platinum-based chemotherapeutics by decreasing β-catenin. Therefore, KAT6A inhibition may be a potential therapeutic strategy in ovarian cancer treatment.

## Discussion

**Figure 7:**
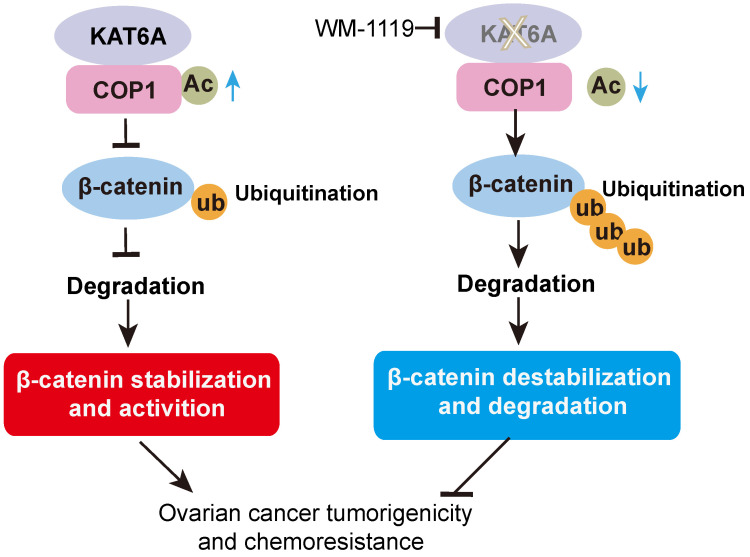
Proposed model underlying the roles of KAT6A-mediated COP1 acetylation in ovarian cancer.

In this study, we discovered a new KAT6A-COP1-β-catenin oncogenic axis that promoted tumorigenicity and the response to cisplatin in ovarian cancer. KAT6A was significantly upregulated in ovarian cancer and correlated with poor survival in ovarian cancer patients. COP1 acetylation by KAT6A decreased the ubiquitination of β-catenin, leading to enhanced β-catenin stability. Pharmacologic inhibition of KAT6A using WM-1119 markedly enhanced the antitumor activity of cisplatin when applied to orthotopic ovarian cancer xenograft models (Figure 7).

Our data demonstrate that KAT6A is critical for ovarian cancer. Previous studies have suggested that KAT6A plays an oncogenic role in breast cancer 16 and leukemia 18-19. Recently, we demonstrated that KAT6A promotes glioma tumorigenicity by acetylating H3K23 17. In this study, our data indicate that KAT6A is upregulated in ovarian cancer cells and tissues. High KAT6A protein expression correlates with a poor prognosis. Repression of KAT6A resulted in attenuated cell proliferation, invasion and metastasis *in vitro* and xenograft tumor growth. Importantly, high KAT6A expression correlates with a poor prognosis in patients treated with platinum. Genetic or pharmacologic inhibition of KAT6A enhanced ovarian cancer cell response to cisplatin, suggesting that KAT6A inhibition can sensitize ovarian cancer cells to platinum-based chemotherapeutics. Our study provides the theoretical basis for the application of targeting KAT6A in cancer therapy. But the effective concentrations of WM-1119 were different in various tumours due to heterogeneity of different tumours 22. Further optimization of specificity and efficiency is needed for the application of KAT6A inhibitors.

KAT6A has been reported to acetylate both nonhistone and histone substrates in mammals 11,21. In PML, KAT6A acts as an acetyltransferase of p53 at K120 and K382 and enhances p53-dependent p21 expression 14,21. Here, we identified COP1 as a new nonhistone substrate of KAT6A. COP1, also called RFWD2, possesses a RING finger domain and functions as an E3 ubiquitin ligase 32 and is highly conserved among various vertebrates 33. COP1 was initially identified as a central negative regulator of photomorphogenesis in *Arabidopsis thaliana* 34. Human COP1 promotes the degradation of various targeted proteins by enhancing their ubiquitination, such as p53 35, c-JUN 36, p27, STAT3, ETS2 and β-catenin 27. COP1 participates in regulating cell growth, apoptosis and DNA damage repair by targeting its substrates for ubiquitination-mediated degradation 37,38. However, the posttranslational modification of COP1 remains elusive. Here, our data identify that COP1 is a new KAT6A substrate, which advanced our knowledge of the function of KAT6A and COP1 and their association with ovarian cancer.

β-catenin, which is encoded by the CTNNB1 gene in humans, is the key regulator of the Wnt pathway 39,40. Accumulating evidence has stated that aberrant activation of Wnt/β-catenin signaling contributes to various types of malignancies, including breast 41, lung 42, gastric 43, hepatocellular 44, colorectal 45, prostate 46, bone 47, and ovarian 30 cancers. Increased activation of the Wnt/β-catenin pathway plays a critical role in the initiation and progression of cancers by maintaining cancer stem cells, regulating cell proliferation, polarity, invasion, chemoresistance or inhibiting apoptosis 48. Hyperactivation of β-catenin is commonly observed in ovarian cancer. Moreover, several reports have indicated that downregulating Wnt/β-catenin signaling mitigates the malignant behavior of cancer cells, highlighting the potential of the Wnt/β-catenin pathway as a therapeutic target 49. Ubiquitination is one of the main regulatory mechanisms of β-catenin. In the canonical Wnt signaling pathway, β-catenin interacts with the APC complex and is ubiquitinated by the E3 ligase β-TrCP after phosphorylation by GSK3β 31. Recently, β-catenin has been reported to be ubiquitinated by other E3 ligases, such as TRIM33 50, Jade-1 51, Siah-1 52 and c-Cbl 53. Here, the present study has identified that KAT6A-mediated COP1 acetylation at K294 is a critical post-translational modification that maintains β-catenin stability (Figure 5). Acetylation of COP1 by KAT6A impaired β-catenin ubiquitination, which led to the accumulation of the β-catenin protein and its increased transcriptional activity (Figure 5). Therefore, we concluded that KAT6A could promote activation of the Wnt/β-catenin pathway by acetylating COP1 and further promote tumorigenesis, cancer invasion and chemoresistance in ovarian cancer.

## Conclusion

In summary, our work provides new insights into the posttranslational modification of COP1 and β-catenin in ovarian cancer and highlights that KAT6A functions as an acetyltransferase of nonhistone proteins, by which KAT6A is implicated in tumor progression. Additionally, functional experiments uncovered an oncogenic role of KAT6A, which acts as a key factor that links the platinum response to tumor spread through the stabilization of β-catenin protein. These results reveal the intriguing possibility of combining platinum-based treatments with KAT6A inhibition to overcome platinum resistance and prevent ovarian cancer.

## Supplementary Material

Supplementary figures and tables.

## Figures and Tables

**Table 1:** Quantification of KAT6A in ovarian cancer tissues and normal ovarian epithelium tissues. Statistical analyses were performed with the χ^2^ test, *P*<0.05.

	KAT6A^Low^	KAT6A^High^	Total
Normal	6	2	8
Carcinoma	10	30	40
