# Supplementary material for: KAT6A, a novel regulator of β-catenin, promotes tumorigenicity and chemoresistance in ovarian cancer by acetylating COP1
Source: Theranostics. 2021 Apr 15;11(13):6278–92. doi: 10.7150/thno.57455 (PMC8120227; doi:10.7150/thno.57455)
Supplement: Supplementary file 1 — Supplementary figures and tables. [file thnov11p6278s1.pdf]

Supplementary Table 1 Top 20 of identified proteins associated with KAT6A

prot_acc	prot_acc_name	gene name	prot_cover (%)
Q8NHY2	COP1_HUMAN	COP1	21
P10809	CH60_HUMAN	HSPD1	20.1
Q8NFW8	NEUA_HUMAN	CMAS	19.4
Q5QNW6	H2B2F_HUMAN	HIST2H2BF	19
P42575	CASP2_HUMAN	CASP2	18.8
Q6PJ69	TRI65_HUMAN	TRIM65	18.6
Q9BTY7	HGH1_HUMAN	HGH1	17.9
O95801	TTC4_HUMAN	TTC4	17.8
Q13362	2A5G_HUMAN	PPP2R5C	17.4
Q5T2D3	OTUD3_HUMAN	OTUD3	17.3
Q9UJU6	DBNL_HUMAN	DBNL	17.2
Q8TBX8	PI42C_HUMAN	PIP4K2C	17.1
P31939	PUR9_HUMAN	ATIC	17.1
Q9BQG2	NUD12_HUMAN	NUDT12	16.9
Q6Y288	B3GLT_HUMAN	B3GLCT	16.5
Q9Y4P1	ATG4B_HUMAN	ATG4B	16.3
Q86U42	PABP2_HUMAN	PABPN1	16.3
P35659	DEK_HUMAN	DEK	15.7
Q12874	SF3A3_HUMAN	SF3A3	15.6
P0DOX5	IGG1_HUMAN	IGG1	15.4

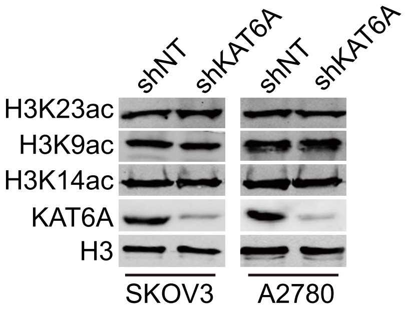

Figure S1 Western blot analysis of the effects of *KAT6A* knockdown on the H3K23ac, H3K9ac, and H3K14ac protein levels in SKOV3 and A2780 cells.

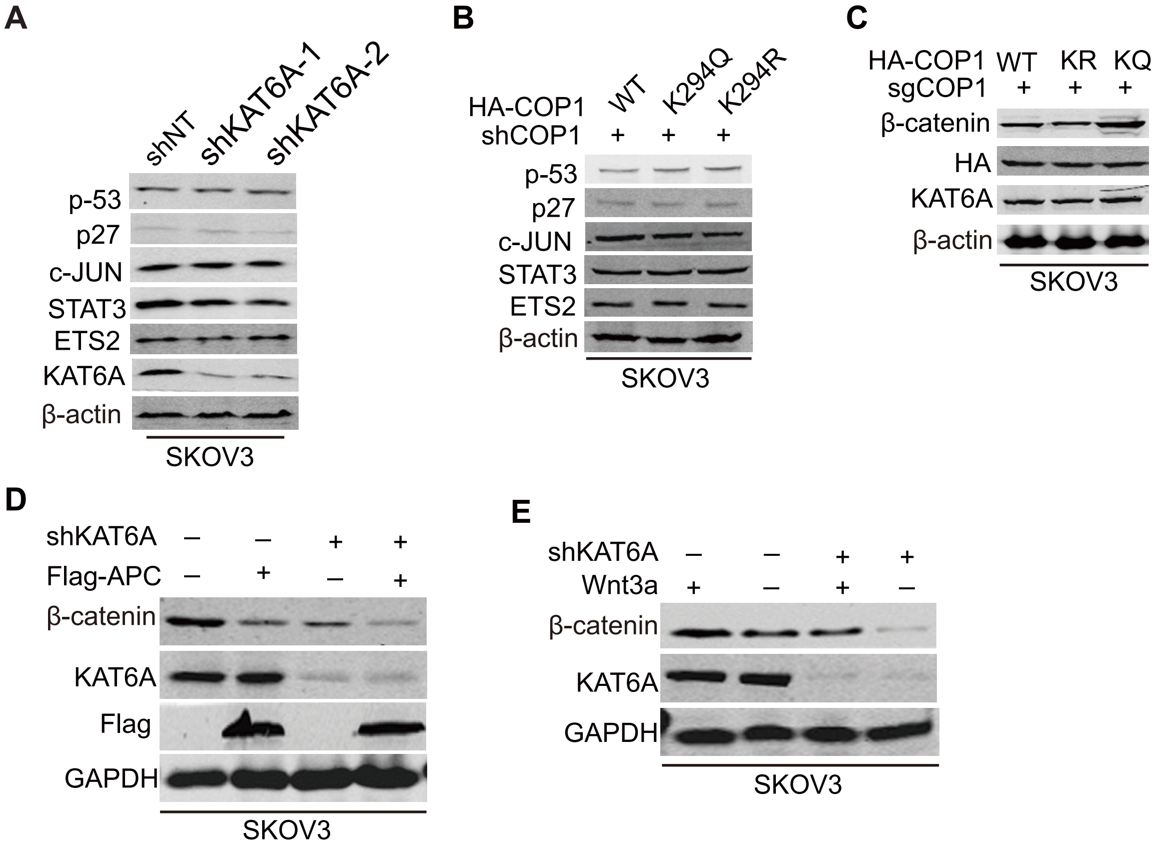

Figure S2 Effects of *KAT6A* KD or COP1 acetylation on β-catenin-targeted downstream protein expression

(A) WB of the effects of KAT6A KD on p53, p27, ETS2, c -JUN, and STAT3 protein levels. **(B)** WB of the effects of COP1 acetylation on p53, p27, ETS2, c -JUN, and STAT3 protein levels. **(C)** Effects of overexpression of shRNA-resistant COP1-WT or COP1-K294Q/K294R mutant on β-catenin protein levels. **(D** and **E)** WB analysis of the effects of KAT6A KO and APC or Wnt3a (100 ng/ml) on β-catenin protein levels.

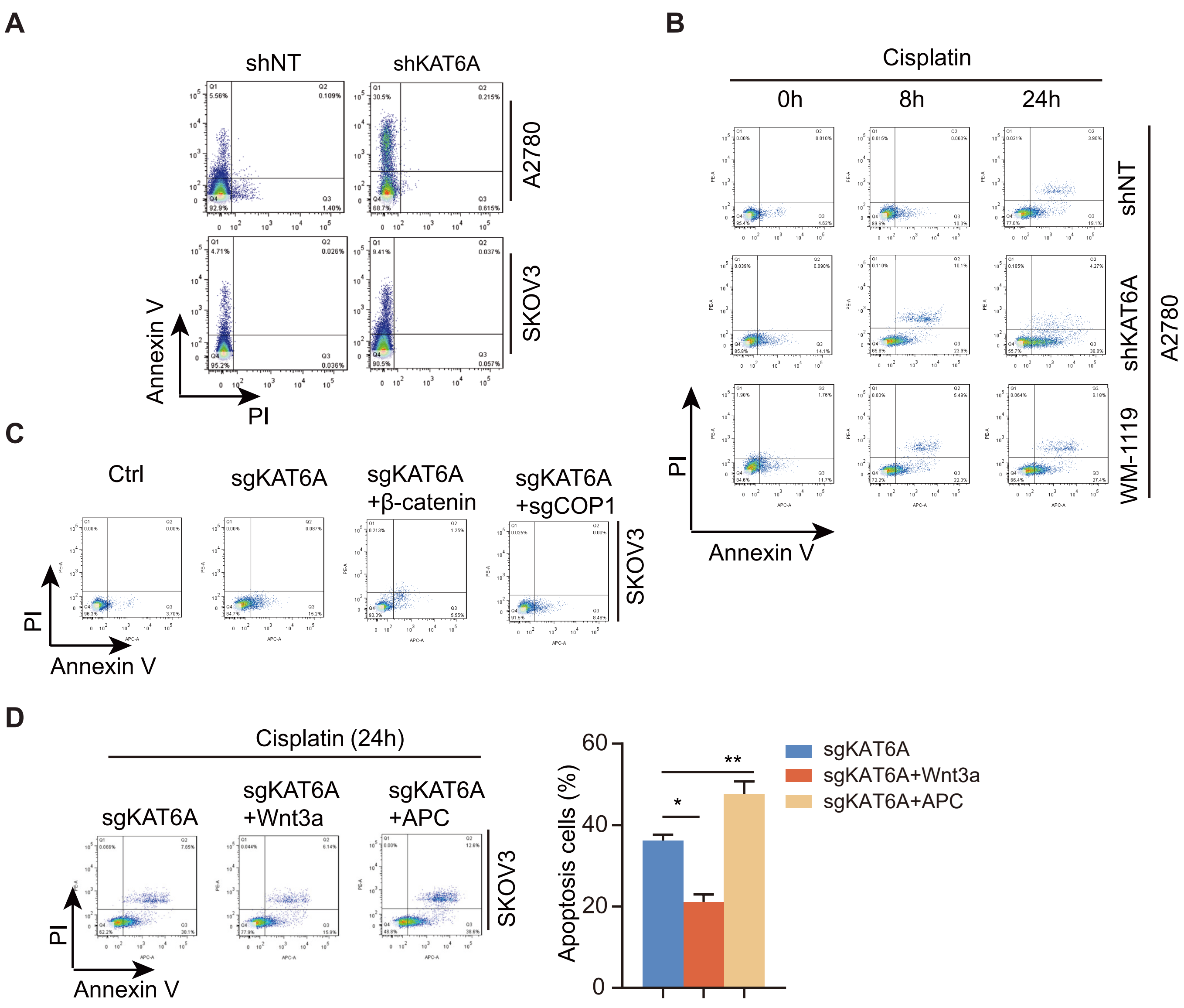

Figure S3 Effects of KAT6A KD on apoptosis

(A) Effects of *KAT6A* KD on apoptosis in SKOV3 and A2780 cells . **(B)** Representative images of apoptosis assays in Figure 6E. **(C)** Representative images of apoptosis assays in Figure 6G. **(D)** Representative images (left) and quantification (right) of apoptosis assays in KAT6A KO SKOV3 cells treated with cisplatin for 24 h with or without Wnt/APC. *$P < 0.05$, **$P < 0.01$.
